# Nonthrombotic internal jugular venous stenosis may facilitate cerebral venous thrombosis

**DOI:** 10.1111/cns.13719

**Published:** 2021-08-16

**Authors:** Xiaoqin Wu, Jingyuan Ya, Da Zhou, Yuchuan Ding, Xunming Ji, Ran Meng

**Affiliations:** ^1^ Department of Neurology Xuanwu Hospital Capital Medical University Beijing China; ^2^ Advanced Center of Stroke Beijing Institute for Brain Disorders Beijing China; ^3^ Department of China‐America Institute of Neuroscience Xuanwu Hospital Capital Medical University Beijing China; ^4^ Division of Clinical Neuroscience University of Nottingham Nottingham UK; ^5^ Department of Neurosurgery Wayne State University School of Medicine Detroit MI USA

**Keywords:** cause, cerebral venous thrombosis, internal jugular venous stenosis, relationship

## Abstract

**Aims:**

To explore the effect of nonthrombotic internal jugular venous stenosis (IJVS) exerted on cerebral venous thrombosis (CVT).

**Methods:**

Patients with imaging confirmed CVT were enrolled into this real‐world case–control study consecutively from January 2018 through April 2021, and were divided into CVT and IJVS‐CVT groups, according to whether or not with non‐thrombotic IJVS. Chi‐square and logistic regression models were utilized for between‐group comparison of thrombotic factors.

**Results:**

A total of 199 eligible patients entered into final analysis, including 92 cases of CVT and 107 cases of IJVS‐CVT. Chi‐square revealed that thrombophilic conditions were found in majority of CVT, while only minority in the IJVS‐CVT group (83.7% vs. 20.6%, *p* < 0.001). Multivariate logistic regression indicated that most identified thrombophilia were negatively related to IJVS‐CVT (all *p* < 0.05), including oral contraceptive use (*β* = −1.38), hyperhomocysteinemia (*β* = −1.58), hematology (*β* = −2.05), protein C/S deficiency (*β* = −2.28), connective tissue disease (*β* = −1.18) and infection (*β* = −2.77). All recruited patients underwent standard anticoagulation, 10 cases in IJVS‐CVT group also received jugular angioplasty for IJVS correction. Most participants obtained alleviations during 1‐year follow‐up. However, both clinical and imaging outcomes in IJVS‐CVT group were not as good as those in CVT group (both *p* < 0.05). Moreover, 8 cases with CVT and 7 cases with IJVS‐CVT were rehospitalized for CVT recurrences and underwent customized treatment.

**Conclusion:**

Nonthrombotic IJVS may be one of the risk factors of CVT. Anticoagulation might need to be suggested for IJVS patients.

## Graphical abstract

Nonthrombotic internal jugular vein stenosis (IJVS) may predispose to cerebral venous thrombosis (CVT) formation through the obstructed venous outflow and hemodynamic changes, which could be supported by our results: firstly, a vast majority of simple CVT patients had thrombosis‐prone conditions, while such a situation only accounted for a minority in patients with CVT combined nonthrombotic IJVS; secondly, CVT relapse occurred in 8 cases with inherited thrombophilia in simple CVT group, and in 7 cases without any thrombophilic conditions in the IJVS‐CVT group, indicating that nonthrombotic IJVS could possibly promote the CVT recurrence like inherited thrombophilia did. Besides, nonthrombotic IJVS may be the candidate for anticoagulation and stenting to prevent CVT formation, although clinical trials with large simple size are still needed.

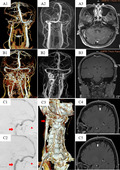

## 1 INTRODUCTION

Cerebral venous thrombosis (CVT) refers to the thrombotic steno‐occlusion of dural sinuses and/or cerebral veins, mainly affecting the young and middle‐aged adults with a particular predilection for females.^1^ Patients with CVT usually present with a wide constellation of nonspecific symptoms, such as headache, blurred vision, tinnitus, etc. Note that various risk factors may predispose to CVT, including infective or noninfective, and inherited or acquired.^2^ Or alternatively, the etiologies that mediated the CVT formation can also be categorized according to the classic Virchow triad, that is, endothelial damage, blood hypercoagulability or hemodynamic changes.^3^ Although the increasing availability of modern neuroimaging modalities has advanced the early recognition of CVT with highly decreased mortality,^4^ CVT misdiagnosis and missed diagnosis still remain as main issues in clinical settings at present, owing to its nonspecific manifestations and various causes. Such situations place the patients at an increased risk of long‐lasting illness, treatment delay, and even repeated relapse.^5^ However, it is of utmost necessity to detect CVT and its origins as well as to explore the potential causes of CVT besides the well‐known etiologies.

Notably, one prior study conducted by our team demonstrated that the narrowing internal jugular vein (IJV), detected by color Doppler flow imaging (CDFI), may disturb intracranial venous hemodynamics and therefore promote CVT occurrence.^6^ However, as we all know that the values of CDFI may be varied from different operators for its operation dependence. Herein, we aimed to further establish the association between nonthrombotic IJVS and CVT based on neuroradiological imaging evidence to provide references for customized treatment and spark future studies with prospective directions.

## 2 MATERIAL AND METHODS

### 2.1 Patient enrollment

Patients with magnetic resonance black‐blood thrombus imaging (MRBTI) confirmed CVT were enrolled into this real‐world case–control study consecutively from January 2018 through April 2021 in Xuanwu Hospital, Capital Medical University. Considering that IJV thrombosis (IJVT) may develop as a result of CVT extension,^5^ or perhaps occur spontaneously with CVT as triggered by the same pathology,^7^ cases with MRBTI‐confirmed IJVT were rejected from this study. All eligible patients signed the informed consents prior to enrollment and data acquisition.

Demographics, clinical and neuroimaging data of all patients were obtained from our inpatient database. The conditions of bilateral IJVs in all patients enrolled were assessed into normal or stenotic conditions, according to the maps of head‐and‐neck contrast‐enhanced magnetic resonance venography (CE‐MRV) and/or computerized tomography venography (CTV). Premised on the results mentioned above, patients were divided into CVT group (isolated CVT without nonthrombotic IJVS) and IJVS‐CVT group (CVT combined with unilateral or bilateral nonthrombotic IJVS). Worthy of note is that IJVS is defined as the inner diameter at the stenosis segment less than 50% of the one at the adjacent normal segment, accompanied with abnormal collaterals.^8^, ^9^

All patients were strictly selected with the following inclusion and exclusion criteria:

Inclusion criteria: (a) imagi
ng confirmed isolated CVT, or CVT coexisted with nonthrombotic IJVS; (b) no age and gender restrictions; (c) complete clinical and imaging data.
Exclusion criteria: (a) MRBTI‐confirmed IJVT; (b) any other conditions that may influence intracranial pressure (ICP), such as arteriovenous fistula, cerebral space‐occupying lesions, idiopathic intracranial hypertension (ICH) and so on; (c) psychiatric disorders or other severe systematic illnesses; (d) incomplete clinical or radiological information.

### 2.2 Clinical data collection and evaluation

Both diplopia and visual field defects were inducted into the category of visual disorders in this study. Ophthalmologists with extensive experience performed color fundus photography for all patients enrolled. The intensity of papilledema was graded using the Frisén scale. The Frisén value ranges from a minimum score of 0 to a maximum of 5, and the severer papilledema is awarded with the correspondingly higher Frisén score.^10^ In addition, details regarding the status of optic nerve sheath fenestration (ONSF) were gathered from the electronic medical records. Almost all participants underwent lumbar puncture, by which, ICP was measured and cerebrospinal fluid (CSF) opening pressure of 200 mmH_2_O or more was identified as ICH.^11^ As restricted by the ICP monitoring tube, the upper limit of CSF pressure was 330 mmH_2_O. All CVT patients who fulfilled the screening criteria were included in the final analysis, whether or not with clear etiologies. Referring to literatures about the CVT etiologies,^2^, ^12^, ^13^, ^14^ well‐established risk factors of CVT in this study mainly covered with overweight (body mass index, BMI > 25.0 kg/m^2^), obstetric causes (pregnancy or postpartum), oral contraceptive use, hemopathy (i.e., anemia, thrombocythemia or polycythemia vera), protein C/S deficiency, nephrotic syndrome, connective tissue disease (i.e., antiphospholipid syndrome, APS and systemic lupus erythematosus, SLE), hyperhomocysteinemia (>20.0 mmol/L), and infection (i.e., mastoiditis, periodontal abscess, etc.) as well as other causes (such as diarrhea, therapy with cyclosporine A, malignancy, ovarian hyperstimulation syndrome, hormonal therapy, etc.). Similarly, such thrombophilic assessments were achieved according to baseline check‐ups and electronic medical records at admission.

### 2.3 Neuroimaging assessment

Imaging spectrums of all patients enrolled were retrospectively reviewed from medical imaging system to validate the nonthrombotic IJVS and preclude the MRBTI‐diagnosed IJVT or any conditions that might affect ICP. In our institution, magnetic resonance imaging (MRI) scanning was routinely performed in all patients with CVT except for those with claustrophobia and MR contraindications, based on which, the parenchymal lesions, such as venous ischemia or hemorrhagic transformation, were disclosed. A part of patients, especially the candidates for endovascular procedure, were also imaged with other diagnostic modalities, including digital subtract angiography (DSA). It was worth noting that all patients were positioned supine and their head were in neutral positions when being imaged.

**FIGURE 1:**
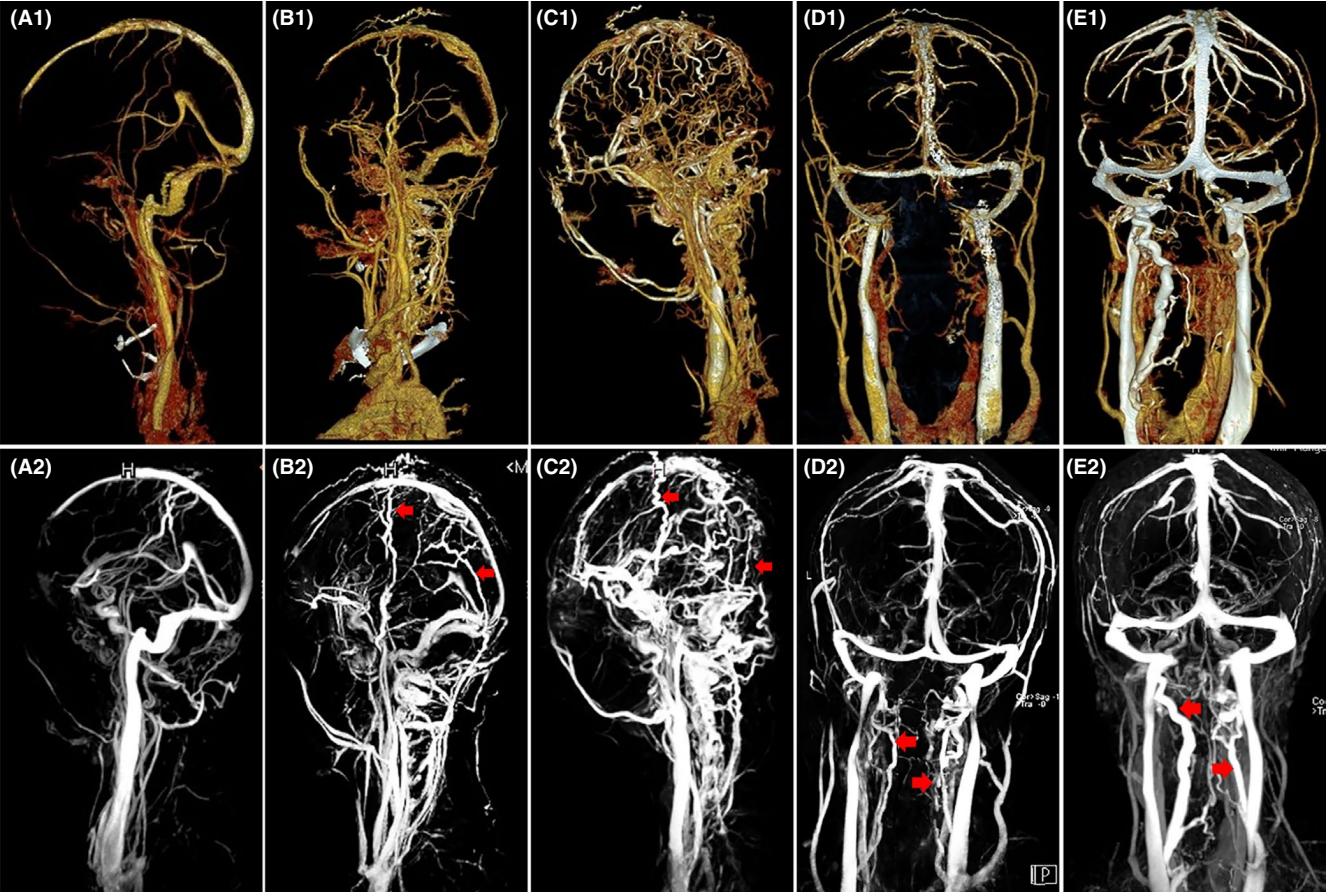
Different patterns of the intra‐ and extracranial collaterals. A1 to E1 were the CTV maps and A2 to E2 were the CE‐MRV pictures. The first three columns: the dilation patterns of the scalp veins (red arrows), no presence (A1and A2), mild dilation (B1 and B2) and severe dilatation (C1 and C2). The other two columns: the dilation patterns of the vertebral collaterals (red arrows), mild degree (D1 and D2) and severe degree (E1 and E2)

Locations (transverse sinus, sigmoid sinus, superior or inferior sagittal sinus, straight sinus or cortical veins) and laterals (right, left or bilateral) of the thrombi in cerebral venous system were confirmed by MRBTI. In cases with CVT plus nonthrombotic IJVS, the stenotic types of IJVS (venous wall damage or external compression) and the patterns of extrinsic compression (osseous, arterial or surrounding tissue compression) were also marked according to the results of CE‐MRV and/or CTV. Furthermore, to determine the relationship between ICH and accessory venous circulation, the intra‐ and extracranial collaterals were evaluated as well, and the extents of which were allocated as mild or severe degrees based on the venograms. In detail, the scoring scheme for dilated scalp veins, namely additional routes for intracranial venous drainage in regions with venous blockage, were assigned as no presence, mild dilation or severe dilatation through visual assessment.^15^ Besides, the dimensions of extracranial vertebral collaterals were classified into mild or severe degrees. The former means the maximal cross‐sectional area of the collaterals <25% of the adjacent normal IJV, while the latter ≥25%.^8^, ^9^ An illustrated map concerning the compensatory patterns of the intra‐ and extracranial collaterals was shown as Figure 1. Two experienced neuroradiologists who were blinded to our clinical data and study intention completed all neuroimaging assessments. The interobserver divergencies were resolved through a discussion, or perhaps, a third rater would persuade the two assessors and decide the final result if the discussion failed to reach a consistency.

### 2.4 Follow‐up study

All patients informed consent to this study were followed‐up by outpatient clinic or detailed telephone‐based interviews. Also, some patients with recurrent CVT were rehospitalized for optimized etiological therapy, which enabled us to access their clinical and neuroimaging data. The follow‐up investigation was undertaken to assess their posttherapeutic conditions through clinical and imaging analysis. Clinical follow‐up duration equaled to the time interval from the first hospitalization to outpatient clinic or rehospitalization. The period of imaging follow‐up was calculated based on the time span between the baseline and repeated imaging. Clinical outcomes were graded as improvement, no change or recurrence. Correspondingly, neuroimaging outcomes of CVT were assigned based on the recanalization degree as being improved, no change or worsen.

### 2.5 Approvals and consents

This study was approved by the Institutional Ethic Committee of Xuanwu Hospital, Capital Medical University, Beijing, China. The consent forms of all patients were signed prior to information collection.

### 2.6 Statistical analysis

Statistical Package for Social Sciences (SPSS) software version 25.0 was applied for our data processing. Numerical variables following the normal distribution were expressed as mean ± standard deviation (M ± SD) and calculated by two independent samples *T*‐test; otherwise, they were presented as median (interquartile range, IQR) and analyzed by the Mann–Whitney U test. Categorical values were described as numbers (percentages) and examined by Pearson Chi‐square test or Fisher's exact tests. For further analysis, binary logistic regression model was built up to examine the confounding effects of thrombophilia conditions imposed on IJVS‐CVT. And the results were displayed as estimated *β* and odd ratio (OR) along with 95% confidence interval (95%CI). Furthermore, Stata 12.0 software was implemented to analyze the correlation and to construct the forest plots. Statistical significance was established at two‐tailed value of *p* < 0.05.

## 3 RESULTS

### 3.1 Demographics

A total of 199 patients with MRBTI confirmed CVT were enrolled consecutively from January 2018 through April 2021, and were grouped into CVT (*n* = 92) and IJVS‐CVT (*n* = 107) groups, depending on the absence or presence of nonthrombotic IJVS. Details were displayed in Table 1. No statistical differences were found in the gender ratio and BMI between the CVT group (M/F = 41/51, 25.7 ± 4.6 kg/m^2^) and IJVS‐CVT (M/F = 44/63, 24.8 ± 3.6 kg/m^2^) group, both *p* > 0.05. However, the age difference between the two groups reached remarkable statistical significance (CVT vs. IJVS‐CVT = 37.4 ± 13.3 vs. 45.2 ± 14.7 years old, *p* < 0.001).

**TABLE 1:** Clinical features in CVT patients with normal or stenotic IJV

Items	CVT	IJVS‐CVT	*p*‐value
Demographics			
Case numbers (n)	92	107	NA
Gender (male/female)	41/51	44/63	0.624
Age, year (M ± SD)	37.38 ± 13.25	45.19 ± 14.72	<0.001*
BMI (kg/m^2^)	25.68 ± 4.58	24.79 ± 3.64	0.134
Symptoms, n (%)			
Headache	81 (88.0)	64 (59.8)	<0.001*
Nausea and vomiting	51 (55.4)	31 (29.0)	<0.001*
Visual disorders	28 (30.4)	14 (13.1)	0.003*
Visual decline	40 (43.5)	31 (29.0)	0.033*
Hearing deterioration	4 (4.3)	9 (8.4)	0.247
Head noise	4 (4.3)	25 (23.4)	<0.001*
Tinnitus	24 (26.1)	36 (33.6)	0.247
Dizziness	22 (23.9)	40 (37.4)	0.041*
Neck discomfort	14 (15.2)	18 (16.8)	0.759
Sleep disturbance	16 (17.4)	39 (36.4)	0.003*
Anxiety and depression	8 (8.7)	21 (19.6)	0.029*
Subjective memory decline	8 (8.7)	8 (7.5)	0.753
Seizure	9 (9.8)	16 (15.0)	0.273
Complications, *n* (%)			
Papilledema	58 (63.0)	23 (21.5)	<0.001*
FPG scores, median (IQR)	2.23 (0.0–5.0)	0.60 (0.0–1.0)	<0.001*
Cases with lumbar puncture	83 (90.2)	72 (67.3)	NA
ICH, number (%)	70 (84.3)	41 (56.9)	<0.001*
CSF opening pressure, mmH_2_O (M ± SD)	288.61 ± 65.35	221.04 ± 76.43	<0.001*
Ischemic venous infarction	5 (5.4)	13 (12.3)	0.091
Hemorrhagic venous infarction	6 (6.5)	8 (7.5)	0.763
Comorbidities, *n* (%)			
Hypertension	18 (19.6)	26 (24.3)	0.422
Diabetes mellitus	4 (4.3)	7 (6.5)	0.499
Hyperlipidemia	22 (23.9)	16 (15.0)	0.109
Thrombophilia conditions, *n* (%)			
Free from risk factors	15 (16.3)	85 (79.4)	<0.001*
Identified with risk factors	77 (83.7)	22 (20.6)	<0.001*
Pregnancy/puerperium	4 (4.3)	3 (2.8)	0.556
Oral contraceptive use	11 (12.0)	4 (3.7)	0.029*
Hematologic disorders	24 (26.1)	6 (5.6)	<0.001*
Protein C/S deficiency	30 (32.6)	6 (5.6)	<0.001*
Nephrotic syndrome	6 (6.5)	0 (0.0)	0.007*
Connective tissue disease	11 (12.0)	3 (2.8)	0.012*
Hyperhomocysteinemia	19 (20.7)	4 (3.7)	<0.001*
Infection	11 (12.0)	1 (0.9)	0.001*
Other risks	18 (19.6)	3 (2.8)	<0.001*
Customs and habits, *n* (%)			
Tobacco use	9 (9.8)	12 (11.2)	0.743
Alcohol drinking	14 (15.2)	15 (14.0)	0.811

Note

* indicates statistical significance as *p*‐value < 0.05.

ICH indicates intracranial hypertension; CSF indicates cerebrospinal fluid.

### 3.2 Clinical features

As in Table 1, co‐dominant clinical symptoms between CVT and IJVS‐CVT groups involved: hearing deterioration (4.3% vs. 8.4%), tinnitus (26.1% vs. 33.6%), neck discomfort (15.2% vs. 16.8%), plus subjective memory decline (8.7% vs. 7.5%), and seizure (9.8% vs. 15.0%), all *p* > 0.05. Moreover, the prevalence of head noise, dizziness, sleeping disturbance, anxiety, and depression were more pronounced in IJVS‐CVT than those in CVT (all *p* < 0.05). And further, 83 cases (90.2%) in the CVT group and 72 cases (67.3%) in IJVS‐CVT group underwent lumbar puncture, and the ratio of ICH in the former was obviously larger than that in the later (84.3% vs. 56.9%, *p* < 0.001). Meanwhile, the average CSF opening pressure in the CVT group was higher than that in the IJVS‐CVT group (288.6 ± 65.4 vs. 221.0 ± 76.4 mmH_2_O), and the ICH‐related symptoms and signs showed prominent preponderance in the CVT group over the IJVS‐CVT group (all *p* < 0.05), including headache (88.0% vs. 59.8%), nausea and vomiting (55.4% vs. 29.0%), visual disorders (30.4% vs. 13.1%), eyesight decline (43.5% vs. 29.0%), papilledema ratios (63.0% vs. 21.5%), and Frisén scores (median scores: 2.2 vs. 0.6). Additionally, the incidence of cerebral venous infarctions, irrespective of ischemia or hemorrhage, was mildly higher in the IJVS‐CVT group than that in the CVT group, even though not reaching statistical difference (*p* > 0.05). The history of hypertension, diabetes mellitus, hyperlipidemia, smoking, and alcohol use were balanced between the two groups (all *p* > 0.05).

A vast majority of patients in the CVT group were implicated with clear thrombophilia, while such evidence was only found in minority cases in the IJVS‐CVT group (83.7% vs. 20.6%, *p* < 0.001); 85 out of 107 patients (79.4%) in the IJVS‐CVT group failed to identified with any routine risk factors of CVT, which indicated that IJVS might be closely associated with CVT formation. Specifically, statistical differences reached significant levels in almost all of the CVT risks as aforementioned between the two groups (all *p* < 0.05), aside from obstetric causes (*p* = 0.556). An illustrated diagram was displayed in Figure S1. For further test, as shown in Table 2, a logistical regression model was used to investigate whether the identified CVT etiologies participated in IJVS formation concurrently. Except for overweight and obstetric causes, the correlation analyses indicated that there were no significant relationship between the factors mentioned below and the IJVS in IJVS‐CVT cases, including oral contraceptive use [*β* = −1.38, adjusted OR (95%CI) of 0.25 (0.07, 0.97), *p* = 0.045], hyperhomocysteinemia [*β* = −1.58, adjusted OR (95%CI) of 0.21 (0.06, 0.73), *p* = 0.015], hematologic disorder [*β* = −2.05, adjusted OR (95%CI) of 0.13 (0.05, 0.36), *p* < 0.001], protein C/S deficiency [*β* = −2.28, adjusted OR (95%CI) of 0.10 (0.04, 0.28), *p* < 0.001], connective tissue disease [*β* = −1.18, adjusted OR (95%CI) of 0.20 (0.04, 0.81), *p* = 0.025], and infection [*β* = −2.77, adjusted OR (95%CI) of 0.06 (0.01, 0.57), *p* = 0.014]. Considering the selection bias of this study, such results should be explained, as these thrombophilia profiles infrequently existed in CVT patients that were considered caused by non‐thrombotic IJVS, which was mainly secondary to external compression in this study. A corresponding forest plot is provided as Figure S2.

**TABLE 2:** Logistic regression for assessing risk factors affected IJVS in CVT cohort

Thrombophilia conditions	Univariate analysis			Multivariate analysis		
	*β*	OR (95%CI)	*p*‐value	*β*	OR (95%CI)	*p*‐value
Overweight	−0.21	0.81 (0.47, 1.42)	0.469	−0.43	0.65 (0.32, 1.33)	0.238
Obstetric causes	−0.46	0.64 (0.14, 2.91)	0.559	−0.47	0.63 (0.11, 3.55)	0.595
Oral contraceptive use	−1.25	0.29 (0.09, 0.93)	0.038*	−1.38	0.25 (0.07, 0.97)	0.045*
Hyperhomocysteinemia	−1.90	0.15 (0.05, 0.46)	0.001*	−1.58	0.21 (0.06, 0.73)	0.015*
Hematologic disorders	−1.78	0.17 (0.07, 0.43)	<0.001*	−2.05	0.13 (0.05, 0.36)	<0.001*
Protein C/S deficiency	−2.10	0.12 (0.05, 0.31)	<0.001*	−2.28	0.10 (0.04, 0.28)	<0.001*
Connective tissue disease	−1.55	0.21 (0.06, 0.79)	0.020*	−1.18	0.20 (0.04, 0.81)	0.025*
Infection	−2.67	0.07 (0.01, 0.55)	0.011*	−2.77	0.06 (0.01, 0.57)	0.014*

Note

* indicates statistical significance for *p*‐value < 0.05.

Abbreviations: OR, Odds ratio; CI, Confidence interval.

### 3.3 Neuroimaging findings

As in Table 3, dural sinuses were the frequently vulnerable sites in the CVT group with the following declining order: transverse sinus (87.0%), sigmoid sinus (71.7%), and superior sagittal sinus (46.7%); while in the IJVS‐CVT group, the most susceptible thrombotic location was the cortical veins (69.2%), all *p* < 0.001. In the IJVS‐CVT group, 151 out of the 214 sides of IJVs were stenotic with the involvement of IJV‐J3 (128 sides), J2 (61 sides) or J1 (12 sides) segments. Among which, 41.1% accounted for bilateral, 36.4% involved the left side and 22.4% was at the right side. As regards the stenotic IJVs, 60.9% were observed with external bony compression, 14.6% were squeezed by carotid arteries, 19.9% were considered caused by venous wall contracture, and only 4.6% were pressed by the surrounding tissues (such as muscles, lymph nodes, thyroid).

**TABLE 3:** Radiological features, therapy and follow‐up in CVT patients with normal or stenotic IJV

Items, *n* (%)	CVT (*n* = 92)	IJVS‐CVT (*n* = 107)	*p*‐value
Thrombotic location			
Transverse sinus	80 (87.0)	61 (57.0)	<0.001*
Sigmoid sinus	66 (71.7)	45 (42.1)	<0.001*
Only left side	31 (38.8)	36 (59.0)	NA
Only right side	33 (41.3)	17 (27.9)	NA
Bilateral sides	17 (21.3)	7 (11.5)	NA
Superior sagittal sinus	43 (46.7)	23 (21.5)	<0.001*
Inferior sagittal sinus	4 (4.3)	5 (4.7)	0.912
Straight sinus	13 (14.1)	12 (11.2)	0.536
Cortical veins	33 (35.9)	74 (69.2)	<0.001*
Total stenotic IJV sides	NA	151 (70.6)	NA
Stenotic sides of IJV			
Bilateral IJVS	NA	44 (41.1)	NA
Unilateral IJVS	NA	63 (58.9)	NA
Isolated left IJVS	NA	39 (36.4)	NA
Isolated right IJVS	NA	24 (22.4)	NA
Stenotic segments of IJV			
Segment J1	NA	12 (7.9)	NA
Segment J2	NA	61 (40.4)	NA
Segment J3	NA	128 (84.8)	NA
Stenotic types			
Osseous compression	NA	92 (60.9)	NA
Arterial compression	NA	22 (14.6)	NA
Surrounding muscle or other tissue compression	NA	7 (4.6)	NA
Venous wall damage	NA	30 (19.9)	NA
Intracranial collaterals			
Cases without collaterals	21 (22.8)	60 (56.1)	<0.001*
Cases with collaterals	71 (77.2)	47 (43.9)	<0.001*
Mild scalp vein expansion	32 (45.1)	33 (70.2)	NA
Severe scalp vein expansion	39 (54.9)	14 (29.8)	NA
Extracranial collaterals			
Cases without collaterals	48 (52.2)	0 (0)	<0.001*
Cases with collaterals	44 (47.8)	100 (100.0)	<0.001*
Mild vertebral expansion	37 (84.1)	51 (47.7)	NA
Severe vertebral expansion	7 (15.9)	56 (52.3)	NA
Treatment			
ONSF	26 (28.3)	6 (5.6)	<0.001*
Simple anticoagulant	77 (83.7)	91 (85.0)	NA
Thrombolysis or thrombectomy	4 (4.3)	6 (5.6)	NA
Balloon Angioplasty	1 (1.1)	3 (2.8)	NA
Stenting	6 (6.5)	3 (2.8)	NA
Thrombolysis and stenting	4 (4.3)	1 (0.9)	NA
Balloon dilation and stenting	0 (0)	3 (2.8)	NA
Clinical follow up			
Average follow‐up period (months)	13.0 ± 11.7	12.5 ± 10.4	NA
Improve	78 (84.8)	78 (72.9)	0.042*
No change	6 (6.5)	22 (20.6)	0.005*
Recurrence	8 (8.7)	7 (6.5)	0.566
Imaging follow up			
Cases with follow‐up imaging	42 (45.7)	39 (36.4)	NA
Average follow‐up interval (months)	4.7 ± 6.1	4.4 ± 5.1	NA
Recanalization	30 (71.4)	18 (46.2)	0.021*
No change	8 (19.0)	18 (46.2)	0.009*
Worse	4 (9.5)	3 (7.7)	0.769

Note

* suggests *p*‐value < 0.05.

In terms of collateral venous drainage, expanded scalp veins were discovered in up to 77.2% (71/92) patients in the CVT group, among which, 45.1% (32/71) were mildly and 54.9% (39/71) were severely expanded; yet, in the cohort of IJVS‐CVT, only 43.9% (47/107) individuals harbored scalp venous expansion with 70.2% (33/47) in mild and 29.8% (14/47) in severe level (*p* < 0.001). In addition, the abnormal extended vertebral venous collaterals surrounded the stenotic IJVs were found in all IJVS‐CVT patients (100%) with 47.7% being mildly dilated and 52.3% severely. In contrast, only 47.8% of CVT patients were detected with enlarged vertebral veins with 84.1% in a mild degree and 15.9% in a severe degree (*p* < 0.001). More radiological details were obtainable in Table 3.

### 3.4 Treatment

**FIGURE 2:**
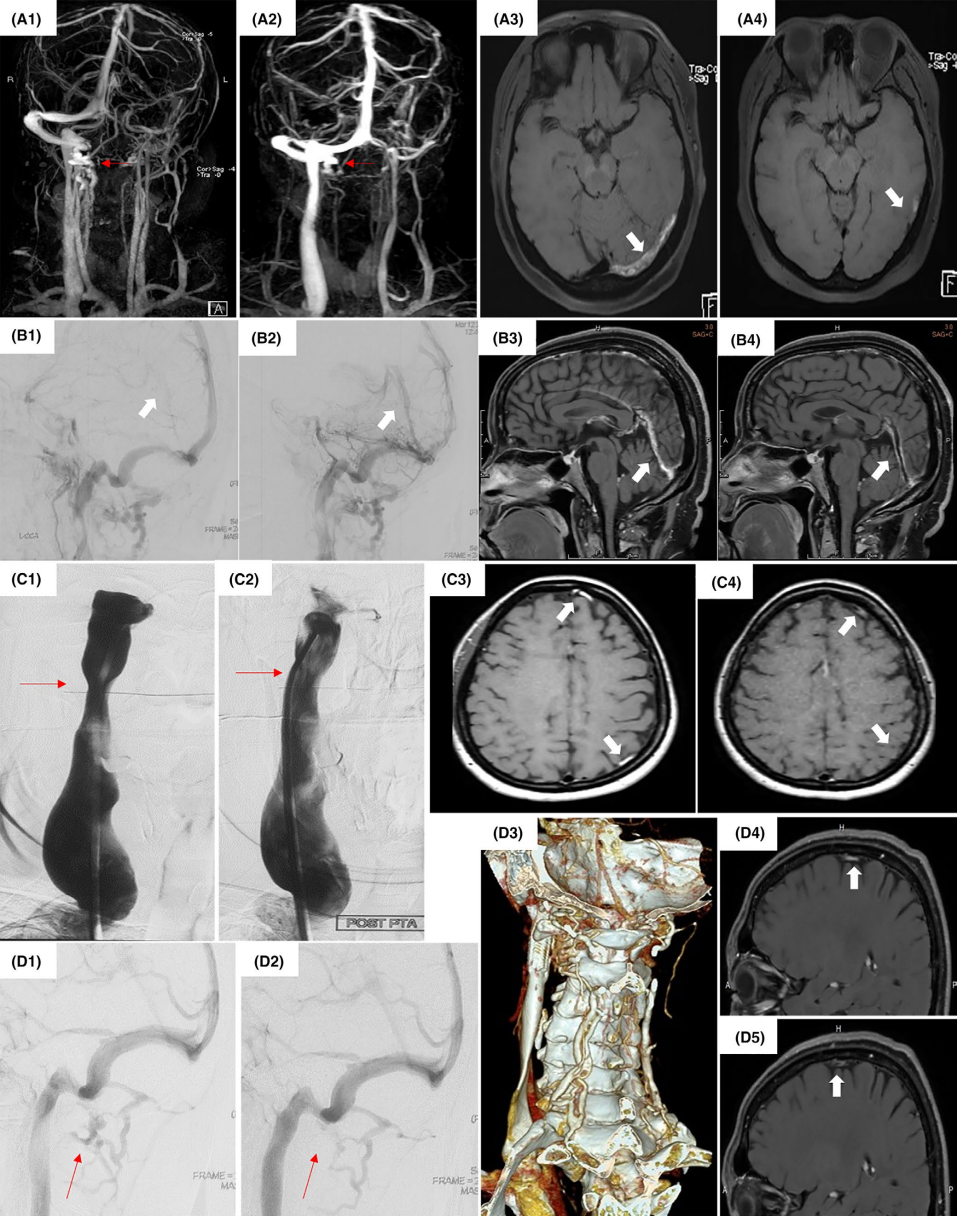
Representative cases pertaining to the treatment patterns. A1‐A4: a CVT patient with mild vertebral dilation was given with oral anticoagulation. The collaterals (red thin arrows) were fewer after (A2) than before (A1) medication, and the thrombi (white arrows) in the left transverse sinus (A3) were almost disappeared after therapy (A4). B1‐B4: a CVT patient with straight sinus thrombosis underwent thrombolysis and mechanical thrombectomy. The straight sinus obtained almost complete recanalization after operation as detected by DSA (B2, white arrow) and MRBTI (B4, white arrow) maps. C1‐C4: an IJVS‐CVT patient underwent IJV balloon dilation. The stenotic IJV was corrected after balloon angioplasty on DSA map (C2, red thin arrow). The baseline vs. follow‐up MRBTI maps (C3 vs. C4, white arrows) revealed the almost completely resolved cerebral cortical venous thrombosis. D1‐D5: an IJVS‐CVT patient underwent IJV stenting. The dilated vertebral veins partly disappeared on the DSA map after IJV correction (D2, red thin arrows). A self‐expanding stent was visible on the CTV image (D3). The baseline vs. follow‐up MRBTI maps (D4 vs. D5, white arrows) revealed that the cortical veins obtained recanalization

As for treatment patterns (Table 3), due to the different CSF opening pressure in the two groups, 26 out of 92 CVT cases (28.3%) received ONSF, whereas only 6 of 107 IJVS‐CVT cases (5.6%) did, *p* < 0.001. All patients underwent standard anticoagulation, among which, 77 cases (83.7%) with CVT and 91 cases (85.0%) with IJVS‐CVT were conservative with isolated anticoagulation without other adjunctive interventions, while 15 cases (16.3%) with CVT and 16 cases (15.0%) with IJVS‐CVT underwent anticoagulation plus endovascular interventional treatment. A case example with CVT resolution after isolated anticoagulation was presented as Figure 2A. Here 4 cases (4.3%) in the CVT group and 6 cases (5.6%) in the IJVS‐CVT group received urokinase‐based catheter thrombolysis and/or mechanical thrombectomy (Figure 2B); 11 cases with CVT and 10 cases with IJVS‐CVT underwent balloon dilation, or venoplasty with self‐expanding stents, or a combination of both so as to restore venous flow (Figure 2C and D).

### 3.5 Follow‐up outcomes

The average duration of clinical follow‐up in this study was 13.0 ± 11.7 months in the CVT group and 12.5 ± 10.4 months in the IJVS‐CVT group (Table 3). During the follow‐up period, 84.8% of CVT and 72.9% of IJVS‐CVT patients obtained symptom alleviations (*p* = 0.042), while 6.5% of the patients in the CVT group and 20.6% in the IJVS‐CVT group reported residual symptoms (*p* = 0.005). Yet, as showed in Table S1, CVT recurrence emerged in 8 cases with isolated CVT in the context of unauthorized discontinuation of anticoagulants or poorly controlled known risk factors. Relapsed CVT also occurred in 7 cases with IJVS‐CVT (among which, 6 cases were without any known risk factors of CVT). Two cases were readmitted for remedial treatment (anticoagulation plus one with balloon angioplasty and another with endovascular stenting), and both of them maintain good conditions at present; the remaining 5 patients with IJVS‐CVT underwent long‐term continuous anticoagulation, and their recurrent symptoms were gradually relieved during the follow‐up time. As the small number of recurred cases, the efficacy of IJVS reconstruction and long‐term anticoagulation in inhibiting CVT relapse in patients with IJVS‐CVT could not be concluded, and our further study is still on going.

**FIGURE 3:**
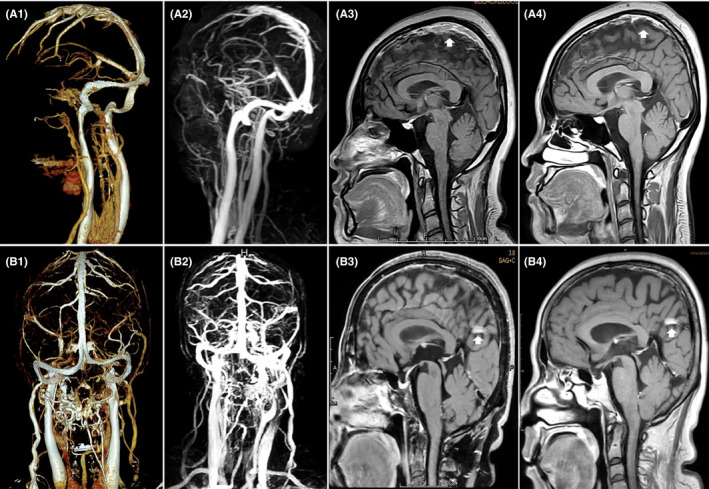
Representative photographs of CVT and IJVS‐CVT. Simple CVT (A1‐A4): CTV (A1) and CE‐MRV (A2) showed no vertebral collaterals around the bilateral normal IJVs. Baseline sagittal MRBTI map (A3) revealed the fresh thrombi (white arrow) within the superior sagittal sinus, which achieved partial recanalization after 7 days of standard anticoagulation, as reflected by follow‐up MRBTI (A4, white arrow). IJVS‐CVT (B1‐B4): CTV (B1) and CE‐MRV (B2) presented the aberrant collaterals surrounded the severe stenotic IJVs. Sagittal MRBTI map discovered the CCVT (B3, white arrow), the sign of which remained not attenuated after 2 months of standard anticoagulation (B4, white arrow)

In the imaging follow‐up, only 42 cases (45.7%) in the CVT group and 39 cases (36.4%) in the IJVT‐CVT group underwent repetitive MRBTI scanning with an average interval of 4.6 months. Compared with their baseline MRBTI maps, 30 patients (71.4%) with CVT and 18 patients (46.2%) with IJVS‐CVT obtained recanalization, *p* = 0.021 (Figure 3A); 8 cases of CVT (19.0%) and 18 cases of IJVS‐CVT (46.2%) showed no remarkable recanalization, *p* = 0.009 (Figure 3B). The thrombi burden in 4 cases in the CVT group with uncorrected causes and 3 cases in the IJVS‐CVT group without any known risk factors were deteriorated (*p* = 0.769). All the results mentioned above revealed that IJVS might not only promote CVT formation but also impede CVT recanalization, despite the short conclusion reliability for the small number of CVT relapse.

## 4 DISCUSSION

Our findings have a directive significance for clinical practice, as coming up with a point of view that IJVS may be a promoting factor for CVT occurrence, which is independent of the currently well‐recognized risk factors. Specifically, both CVT and IJVS may manifest with headache as a corollary of elevated ICP or venous insufficiency, resulting in misinterpretation as either mere CVT or nonthrombotic IJVS in IJVS‐CVT patients. The findings in this study revealed that nonthrombotic IJVS might be a noteworthy danger for CVT, whereby patients with nonthrombotic IJVS should be screened for CVT and vice versa, which is not yet a clinical routine workflow. More importantly, this study indicated that patients with nonthrombotic IJVS might derive benefits from anticoagulation; and IJVS correction might inhibit IJVS‐mediated CVT occurrence and recurrence. Yet, only 10 cases in this study involved with IJVS correction and hence lacked conclusion reliability.

### 4.1 Background differences between CVT and IJVS‐CVT

In this study, thrombophilic conditions were found in the majority of CVT cases (83.7%), whereas confined to a minority in those with IJVS‐CVT (20.6%). For which, we suspected that nonthrombotic IJVS might serve as a primary menace to CVT in patients without recognized thrombotic factors. Owing to the nature of the real‐world case study, we failed to recruit sufficient cases of healthy controls and perform the comparison as to the occurrence of CVT between the large cohorts with and without IJVS. Despite which, a cross‐sectional study with a large sample reported that jugular luminal narrowing could be noted in approximately 36.6% individuals in the general population.^16^ In comparison, the incidence of IJVS in the consecutive entire CVT cohort of this study was 53.8% (107/199). The larger proportion of IJVS in patients with CVT versus those without may indicate a causal connection between IJVS and CVT. However, someone may question the causal relationship between IJVS and CVT explaining that the preponderance of IJVS in CVT individuals may probably be mediated by a third associated factor, such as immunological diseases and hyperhomocysteinemia that can simultaneously work in the formation of IJVS and CVT.^17^ Our study design successfully escaped from such dispute, as the IJVS‐CVT cohort in this study was prevailingly without known CVT‐provoked factors. For further determination, binary logistic regression was conducted and further confirmed that most thrombophilia conditions were negatively associated with IJVS‐CVT. This may be explained that the enrolled IJVS patients in this study were mainly due to the age‐dependent external compression, whereas patients with thrombophilia‐triggered CVT were relatively young and without compressed IJVs.

### 4.2 Probable mechanism for thrombosis in IJVS‐CVT

According to the Virchow triad, entities that are responsible for endothelial dysfunction, blood hypercoagulability, and hemodynamic aberrations are the three elements for venous thrombus formation.^3^ It is well‐known that nonthrombotic IJVS is one of the culprits of venous flow restrictions and even stasis with considerable chronicity. Such venous lesions may contribute to hemodynamic alterations^18^ and thus facilitate CVT generation, even with the absence of endothelial damages or prothrombotic conditions. Of course, disturbed blood flow induced by nonthrombotic IJVS may also function together with other CVT risk factors to provoke CVT, as presented in our dataset. Particularly powerful circumstantial evidence was also obtained in animal studies that unraveled the effect of IJVS‐induced sluggish blood flow imposed in thrombosis genesis using mouse models, which initiated the venous thrombosis through partial‐to‐complete vein ligation without endothelial damage and procoagulant, a mimicker of nonthrombotic venous steno‐occlusion, and concluded that the stenotic degree might positively affect the thrombotic process and clot size.^19^, ^20^ Future studies can explore the accurate pathophysiological mechanisms of IJVS‐induced CVT with assistance from robust imaging modalities, such as multimodal functional MRI (fMRI), arterial spin labeling (ASL), to provide more comprehensive pictures regarding the IJVS‐mediated cerebral hemodynamic changes by better interpreting the status of the whole‐brain cerebral blood flow (CBF).^21^, ^22^, ^23^

### 4.3 Thrombus distribution in CVT and IJVS‐CVT

In this study, MRBTI was employed to display the direct visualization of thrombi.^24^ Thanks to which we found that the thrombi in CVT were often distributed in venous sinuses, and only 35.9% involved cortical veins, namely cerebral cortical venous thrombosis (CCVT); conversely, the CCVT in the IJVS‐CVT group accounted for up to 69.2%. We hence reasoned that nonthrombotic IJVS may probably impart higher thrombotic risk to the cortical veins rather than the venous sinuses, one possible explanation for which is that the impeded venous drainage caused by IJVS may more likely block the distal cerebral venous outflow with the ensuing delayed venous emptying within the distant cortical veins. Such a situation may render the cortical veins fill with static blood thereby contributing to CCVT. Another possible explanation is that IJVS may impair the CBF and therefore decrease the perfusion pressure of the superficial venous system with enhanced venule pressure, thus leading to the formation of CCVT that was strongly related to hypodynamic circulation.^25^, ^26^, ^27^ These explanations may remain undetermined, unless further study is conducted to achieve the precise hemodynamic monitoring in IJVS patients. Also noteworthy is that a recent study has confirmed the involvement of CCVT in the venous infarctions and seizures.^28^ This result was in accordance with the conclusions in our study, as cerebral parenchymal lesions, including ischemia and hemorrhage, along with seizure attacks were more prevailing in the IJVS‐CVT group, despite no statistical significance.

### 4.4 Different symptoms between CVT and IJVS‐CVT

According to our results, the dilated scalp veins and the relevant clinical symptoms, such as headache were more prevalent in CVT patients, whereas older ages, head noise, dizziness, insomnia, in addition to emotion changes and vertebral collaterals were more popular in IJVS‐CVT patients. Correspondingly, the IJVS in this study were mainly caused by the compression of bones or stiffened arteries, both of which were also age‐dependent. The above consisted with a notion that nonthrombotic IJVS tended to be acquired and develop with higher frequency during the aging process as revealed previously.^25^ And the IJVS‐triggered ICH may force the extension of collaterals in order to lighten the drainage burden of the stenotic jugular veins.^29^ During the process of nonthrombotic IJVS, head noise and tinnitus might be deemed as characteristic indicators of IJVS‐induced turbulent flow and the resultant hypoperfusion of the global brain, leading to chronic ischemia of bilateral auditory center and conduction pathways.^25^, ^26^ Meanwhile, sleep and mood disturbances might exist as sequelae of sustained long‐term sufferings. Compared to the CVT group, the milder papilledema and ICH in IJVS‐CVT group could be explained as follows: Firstly, IJVS‐CVT more likely involved the cortical veins rather than dural sinuses and thus imposed little effect on the ICP.^30^ Secondly, preexisting neck collaterals may function as compensatory pathways to relieve the venous pressure and ICP.^29^ Mimic with vertebral collaterals, the existence of highly dilated scalp veins in CVT could be considered as a surrogate marker for anomalous venous return.^31^ Although the dilated scalp veins in CVT provided partial support for the obstructed venous reflow and thereby alleviated the drainage resistance,^15^, ^32^ and might probably decrease the risk of venous infarction,^33^ they were still not strong enough to completely compensate the thrombosis‐mediated ICH. Therefore, the average ICP in the CVT group was obviously higher than that in the IJVS‐CVT group in this study.

### 4.5 Different outcomes between CVT and IJVS‐CVT

During more than 1‐year follow‐up, most patients in the two groups obtained relieves. However, treatment effects on IJVS‐CVT were not as sensitive as those on CVT (20.6% vs. 6.5%, *p* = 0.005). Similarly, compared to baseline MRBTI, the thrombi loaded in 18 cases in IJVS‐CVT remained no remarkable changes in the follow‐up MRBTI maps, while this status was only seen in 8 cases with CVT (*p* = 0.009). For which, we suggested that CVT was triggered by long‐standing IJVS may presumably show more resistance to anticoagulation therapy because of the chronic process of thrombus formation. Of note, our previous work ever highlighted the adjunctive contribution of batroxobin to the dissolution of CVT, which enabled to accelerate thrombolysis and result in rapid recanalization.^34^, ^35^ Although our current study waived to explore the responses of both CVT and IJVS‐CVT to batroxobin, a well‐designed clinical trial regarding batroxobin efficacy in treating CVT and IJVS‐CVT is underway, and will draw a conclusion in the near future.

Aside from the above, CVT relapse occurred in 8 cases with CVT and 7 cases with IJVS‐CVT. The leading causes in the CVT group were the poorly controlled risk factors of CVT plus discontinuation of standard anticoagulants, while in the IJVS‐CVT group, there was nothing except for the blame IJVS plus stopping anticoagulants. For this reason, it is, to some extent, rational to assume that preventive anticoagulation ought to be suggested for IJVS patients, despite not convincing enough. Given that not all participants finished the follow‐up imaging, conclusion regarding the critical role of nonthrombotic IJVS played in CVT prognosis could not be drawn in this study, and the mechanism of which need further clarification.

This is a real‐world case–control study focusing on the role of nonthrombotic IJVS in CVT formation with a relatively large sample size. Previous studies insisted that nonthrombotic IJVS was a normal anatomic variant, as it was highly prevalent without considerable clinical impacts.^36^ A counter‐argument questioning about the effect of IJVS in CVT formation may thus be yielded, minding the fact that most individuals with nonthrombotic IJVS were asymptomatic and not all of them suffered from CVT. Given that, the strict patient selections, including collaterals must present around the deformed IJV and the IJVS without collaterals must be excluded from the participations, were adopted in this study. Noteworthy was that nonthrombotic IJVS with collateral formation was judged to be a pathological entity as previously emphasized.^37^, ^38^ Therefore, we believed that our imaging protocols might act to eliminate the influence from the anatomic jugular variants and the IJVS in this study was indeed a venous anomaly. Besides, our series of studies were already concerned on the relationship between IJVS and CVT based on ultrasound evaluation or case report,^6^, ^39^ and this study further corroborated and extended this finding by incorporating a relatively larger sample, a formal intergroup comparison, multiple neuroimaging evaluation, and long‐term follow‐up analysis.

Current guidelines neither take the nonthrombotic IJVS into account as a risk factor of CVT, nor provide insights into the relevant therapy. In our clinical practice, nonthrombotic IJVS patients were generally screened for CVT and vice versa. Additionally, they underwent long‐term anticoagulation if uncorrected by IJVS operation. Referring to our follow‐up results, the symptoms in most IJVS‐CVT patients were remised and even disappeared after anticoagulation. Besides, IJV correction may be a feasible therapy of choice to prevent CVT recurrence, in view that 10 cases with IJVS‐CVT derived great benefits from the IJVS reconstruction and ultimately obtained good clinical outcomes. However, it should be stressed that the efficacy of IJVS venoplasty in prohibiting CVT recurrence in patients with IJVS‐CVT is somewhat uncertain due to the small sample size of operated cases in this study. Additional studies with larger case numbers and long‐term follow‐up are still warranted.

## 5 LIMITATIONS AND FUTURE DIRECTIONS

Shortcomings are worthy noticing: Firstly, this study is only a mono‐center real‐world case–control study with a relatively short follow‐up period. Secondly, it remains undetermined whether or not nonthrombotic IJVS‐CVT should be granted with long‐term anticoagulation. Thirdly, the cut‐off value of the degree of IJVS on promoting CVT is still not clear. Last but not least, the accurate mechanisms of IJVS‐induced CVT were not elaborated very clearly due to the shortage of auxiliary modalities for a specific hemodynamic study. We hope findings from future studies can shed more light on the questions raised above.

## 6 CONCLUSIONS

To sum up, nonthrombotic IJVS may be a noteworthy risk factor for CVT occurrence, possibly due to the obstructed venous outflow induced by the IJVS. Besides, patients with nonthrombotic IJVS may possibly benefit from continuous anticoagulation and IJVS correction, although further well‐designed clinical trials with a large simple size are still needed to clarify these findings.

## CONFLICT OF INTEREST

XW, JY, DZ, YD, XJ, and RM report no conflicts of interest.

## AUTHOR CONTRIBUTIONS

XW and JY completed the study and drafted this manuscript. RM, DZ, XJ, and YD revised the manuscript and edited English. RM contributed to the conception and design of this study and proposed the amendments.

## CONSENT FOR PUBLICATION

The authors agree to publish.

## Supporting information

Fig S1

Fig S2

Table S1

## Data Availability

Data and materials related to the current study can be accessed from our corresponding author upon reasonable request.
