# Supplementary figures and images for: Nonthrombotic internal jugular venous stenosis may facilitate cerebral venous thrombosis
Source: CNS Neurosci Ther. 2021 Aug 16;27(11):1396–408. doi: 10.1111/cns.13719 (PMC8504525; doi:10.1111/cns.13719)

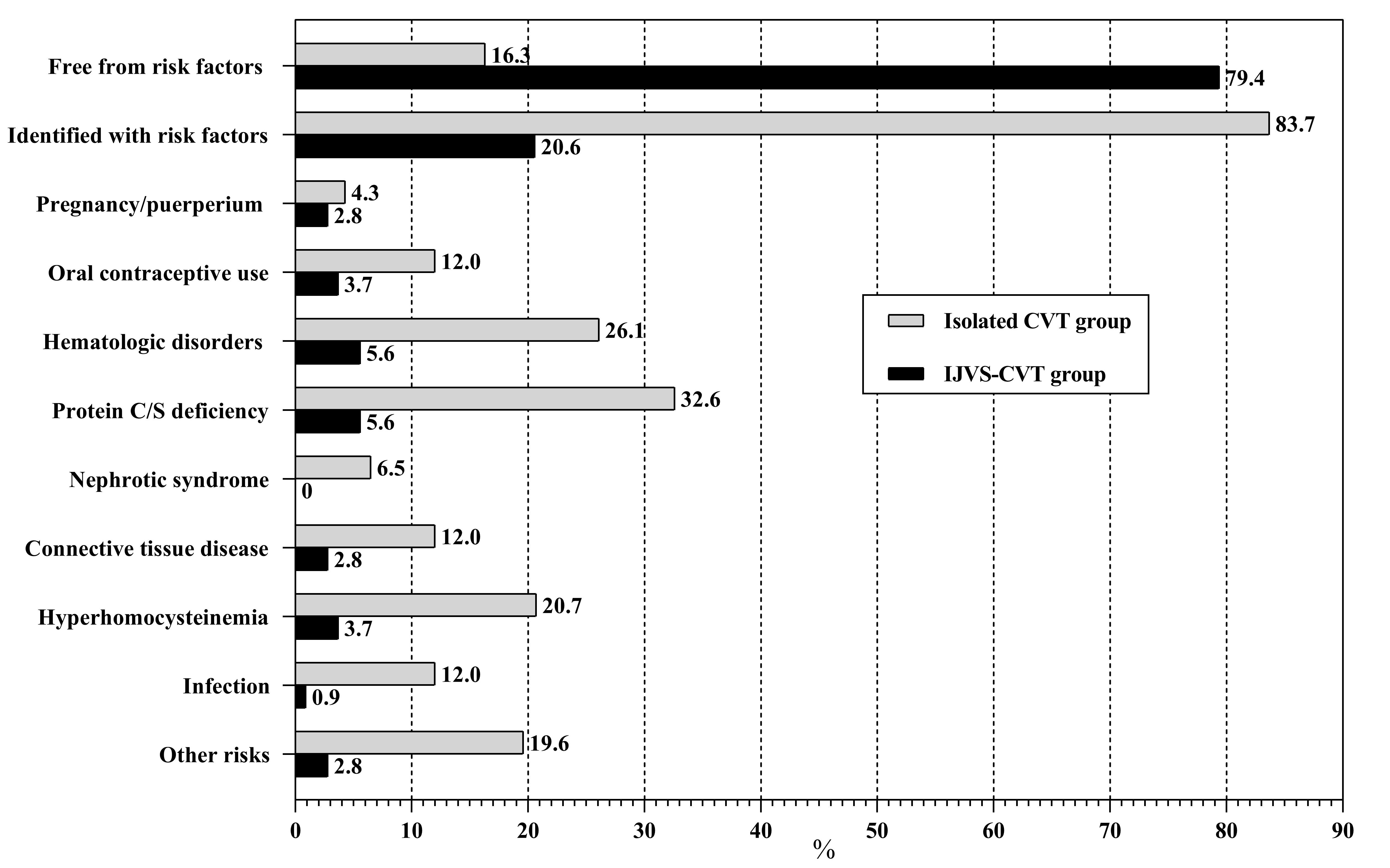

Supplement: Supplementary file 1 — Fig S1 [file CNS-27-1396-s003.tif]

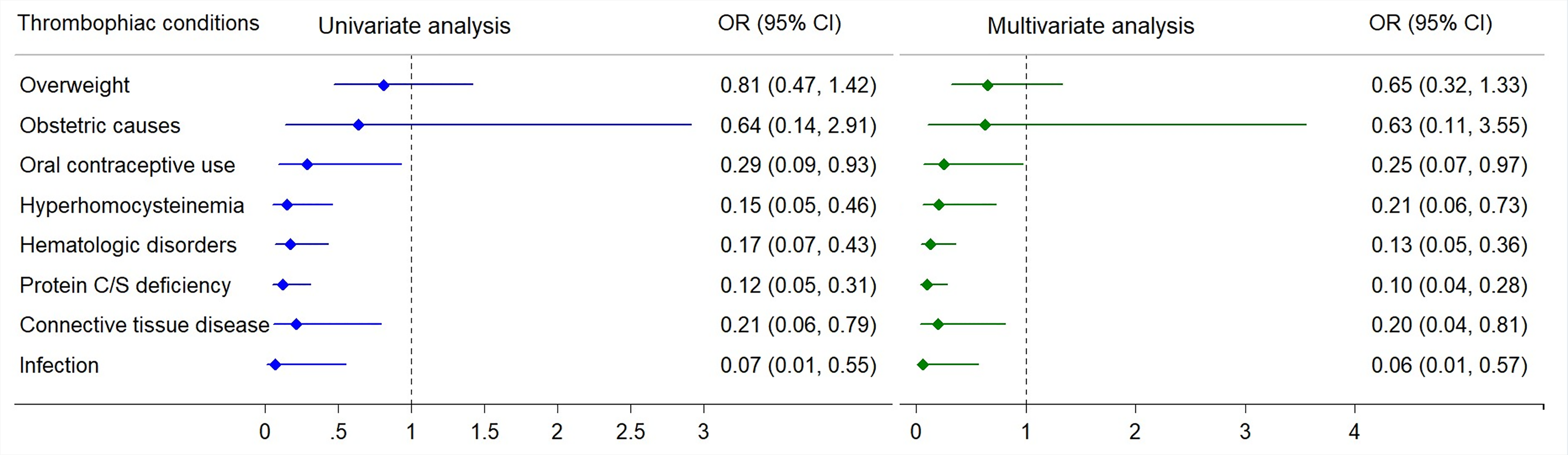

Supplement: Supplementary file 2 — Fig S2 [file CNS-27-1396-s001.tif]
