# Supplementary material for: Nonthrombotic internal jugular venous stenosis may facilitate cerebral venous thrombosis
Source: CNS Neurosci Ther. 2021 Aug 16;27(11):1396–408. doi: 10.1111/cns.13719 (PMC8504525; doi:10.1111/cns.13719)
Supplement: Supplementary file 3 — Table S1 [file CNS-27-1396-s002.docx]

**Table S. Details regarding the cases with CVT recurrence in CVT and IJVS-CVT groups**

| CVT group | |  | | | | | | | |
| --- | --- | --- | --- | --- | --- | --- | --- | --- | --- |
| Number | **Age, years** | **Gender** | **BMI, kg/m^2^** | **Onset symptoms** | **Proposed CVT causes** | **Initial therapy** | **Recurrent interval** | **Recurrent reasons** | **Retreatment** |
| Case 1 | 15 | Female | 29.74 | Headache, vomiting and visual decline | Systemic lupus erythematosus (SLE) | Simple anticoagulation | 3.37 months | Use cyclosporine A (CsA) for SLE | Anticoagulation, replaced CsA with MMF |
| Case 2 | 22 | Female | 33.59 | Headache, nausea, vomiting and seizures | Oral contraceptive use for irregular menstruation | Simple anticoagulation | 15 months | Use the contraceptives again, protein C/S deficiency | Simple anticoagulation |
| Case 3 | 22 | Female | 30.48 | Headache, vomiting and neck pain | Mastoiditis, protein C/S deficiency, OHS and hyperhomocysteine | Urokinase-based catheter thrombolysis | 7 days | Continuous diarrhea and vomiting for three days | Thrombolysis and anticoagulation |
| Case 4 | 24 | Male | 19.15 | Headache, vomiting, visual decline, dizziness, insomnia and neck pain | Primary thrombocythemia due to JAK2V617F mutation | Simple anticoagulation | 8 months | Poorly-managed primary cause | Simple anticoagulation and referred to hematology department for further therapy |
| Case 5 | 31 | Male | 29.00 | Headache, vomiting and dizziness | Nephrotic syndrome | Thrombolysis and anticoagulation | 13 days | Poorly-managed nephrotic syndrome | Thrombolysis, anticoagulation and referred to renal department |
| Case 6 | 42 | Female | 25.39 | Headache and nausea | Thrombocythemia and polycythemia vera | Anticoagulation | 6.43 months | Poorly-managed primary cause | Anticoagulant combined with hydroxyurea and aspirin |
| Case 7 | 43 | Female | 23.11 | Headache, tinnitus and visual decline | Undetected antiphospholipid syndrome (APS) | Anticoagulation | 28 days | Absence of medication for APS | Thrombectomy and balloon dilation combined with APS therapy |
| Case 8 | 53 | Female | 27.73 | Headache, tinnitus and head noise | APS | Thrombolysis | 11.50 months | Discontinuation of anticoagulants | Simple anticoagulant |
| IJVS-CVT group | |  | | | | | | | |
| Case 1 | 13 | Female | 23.95 | Headache, vomiting, depression and neck pain | Anemia due to *ENG* mutation; bilateral IJVS with bony compression | Thrombolysis and mechanical thrombectomy | 11.0 months | Anemia and discontinuation of anticoagulants | Anticoagulant and anemia correction |
| Case 2 | 28 | Male | 22.84 | Headache and dizziness | None, except for slender left IJV and malformed right IJV for bony compression | Anticoagulation | 16.33 months | Discontinuation of anticoagulants | Anticoagulant |
| Case 3 | 34 | Female | 22.31 | Headache, visual decline and insomnia | None, except for the slender upper segments of bilateral IJVs | Anticoagulation | 18.0 months | Discontinuation of anticoagulants | Anticoagulant |
| Case 4 | 46 | Female | 21.83 | Head noise | None, except for the thinness of left IJV and right IJVS for bony compression | Anticoagulation | 28.0 months | Discontinuation of anticoagulants | Anticoagulant |
| Case 5 | 58 | Female | 22.27 | Head noise, tinnitus, and dizziness | None, except for right IJVS for bony compression and the slender left IJVS | Anticoagulation | 22.0 months | Discontinuation of anticoagulants | Anticoagulant |
| Case 6 | 63 | Male | 20.07 | Head noise, tinnitus, dizziness, hearing decline and insomnia | None, except for bilateral IJVS with bony compression | Anticoagulation | 6.0 months | None | Stenting within the right IJV and antiplatelet |
| Case 7 | 64 | Male | 31.60 | Head noise, tinnitus, dizziness, anxiety and insomnia | None, except for left IJVS for bony compression | Anticoagulation | 4.50 months | None | Balloon dilation in the left IJV and anticoagulation |

Notes: SLE indicates systemic lupus erythematosus; CsA indicates cyclosporine A; MMF indicates mycophenolate mofetil; OHS indicates ovarian hyperstimulation syndrome; APS indicates antiphospholipid syndrome.
